# Is Porcelain Inlay Work Impracticable?

**Published:** 1900-11

**Authors:** C. F. Allan


### IS PORCELAIN INLAY WORK IMPRACTICABLE?[1]

BY DR. C. F. ALLAN.

Mr. Chairman,—As I have read the dental literature of the past year and have conversed with my professional friends and acquaintances, it has seemed to me that there is almost a general consensus of opinion in our country, among dentists, that porcelain

[1] Read before The New York Institute of Stomatology, June 5, 1900.

inlay work is impracticable; that at the best it is only available in easily accessible cavities in the buccal and labial faces of the side and front teeth where gold appears in its greatest unsightliness. Few dentists in our country have earnestly advocated its use and very many have decried it; one who has been much in evidence in our journals the past year having even asserted, and seemingly without contradiction, that there has been no such humbuggery perpetrated on the dental profession as that of filling teeth with porcelain.

Now, I do not think there is any gentleman before me who agrees with the dentist I have just quoted, but I wish to tell you of what I saw in Dresden, and I am sure there is no one here to-night who could have seen the results I saw attained in Dr. Jenkins's office and still believe that porcelain inlay fillings are impracticable. Dr. Jenkins is every inch a professional man, and no one here is more in earnest than he is. He has thought over this work and experimented in this line for many years, and now he has his methods in such practical shape that every day I called at his office he had new patients for me to see, a new line of cavities for me to examine. He was most hospitable, and was not only willing but anxious that I should see every step in the process; not only the patient in the chair but also the laboratory work, and I feel that I am greatly indebted to him for his insistence. The patients I saw were not in any way show patients, but were those in every-day practice, with whom in most cases appointments had been made weeks before. I saw all kinds of cavities filled, among them several occlusobuccal cavities in molar teeth as well as proximal cavities in bicuspids, all done with the same seeming ease, with practically little discomfort to the patient, and with the result that the line of demarcation between filling and tooth could hardly be observed.

Dr. Jenkins, in the paper which he read last year before the American Dental Association, said that it was his idea to devise a system, not for the exceptional cases only, *but for the filling of cavities generally as met with in every-day practice, and that was what I saw.* He uses his own low-fusing body, which is the result of an immense amount of experimenting and which seems to me an ideal inlay material. When fused it is homogeneous and can be ground and polished the same as an Ash tooth. It has the strength of any of the high-fusing bodies, and he has made it in most of the shades that will be required. It is very finely ground, and for that reason does not contract to as great an extent as some of the other bodies, and has also the important quality of fusing with exactly the right fluidity, so that any reasonable amount of contour can be attained in its use; this to me is one of its best qualities.

Possibly the feature of Dr. Jenkins's operating that impressed me most was the little attention paid by him to parallelism of walls. Every attention was paid to margins, that they should be sharp and well defined, clean and free from little irregularities, but only as nearly at right angles with the surface of the tooth as was consistent with the easy withdrawal of the matrix, and this latter point was always in view. Retention was then assured by the undercutting of the cavity and the cutting of retaining lines in the sides of the inlay. Every detail seems to have been provided for by him, and small Arkansas stones for the engine, beautifully made and of different shapes, were used for smoothing the margins,—of course this smoothness of wall and freedom from small irregularities made easy not only the withdrawal of the matrix but the future stripping of the gold from the inlay.

Dr. Jenkins has an assistant, and every one who is going to do much inlay work will want an assistant like his. I had the misfortune to break an upper central incisor while in Dresden and sought Dr. Jenkins's services, and he suggested that while I had the space I had better have an unsightly gold filling in the lateral removed and a porcelain inlay put in its place. I feel quite certain that this did not take over three-quarters of an hour. Dr. Jenkins thought best to have two inlays made of different shades to see which would match in color best, his Fräulein making one and he the other. While I was in the chair she came into the room and said she wished to see the tooth to determine how much contour was required, and for such work I could but think that possibly a woman's trained hand would be more delicate than a man's.

A case of restoration I would like to mention as showing the great possibilities esthetically of this work. The lateral incisor of a very comely middle-aged woman had been so mutilated that there was not much more than a peg of a tooth remaining, and this had migrated to the cuspid, so that there was a broad unsightly space between the dwarfed peg of a lateral and the central incisor. He baked and set an inlay on the mesial side of the lateral and then wedged the tooth and inserted an inlay on the distal face, and the transformation in the look of that woman's mouth was simply wonderful; but I think, though, even the patient was not more delighted with the result than I was to have such a perfect standard before me to work up to.

Dr. Jenkins uses a gas furnace. I know that in this country much stress has been put on the advantages of the electric furnace over gas, but I think any one who sees Dr. Jenkins at work in his laboratory and notes the perfect work done so rapidly will feel that he is not working under any great disadvantage. With the high-fusing bodies, however, I imagine the electric furnace is practically a necessity.

In speaking at random in this way I feel that I am not doing Dr. Jenkins justice, but I hope I have convinced you all that inlay work is practical and should be practised, and that all honor is due the deviser of this method, *which instead of showing up decay and announcing defect simulates nature so beautifully.*

In closing a few points occur to me, answers to questions that have been put to me at different times.

The fusing-point of Dr. Jenkins's body I am not certain of, but it is two hundred or three hundred degrees below that of pure gold, a safe margin for the use of the latter as a matrix. He uses numbers 30 and 40 Williams rolled gold, and he takes the matrix direct from the cavity and, as a rule, without the use of the rubber dam.

I have been asked about the amount of space required for the insertion of inlays in proximal cavities, and I can only answer in a general way that the space sufficient for the putting in of a well-contoured gold filling seems sufficient to Dr. Jenkins for the inlay. In baking the inlays it is a general idea that great care should be taken in the cooling off; this is not so, and it is the only point of procedure in which any amount of recklessness can be indulged in. Preferably this work should have a separate room, or at least a corner and a table devoted to it alone, with every convenience, and the greatest attention must be paid to neatness and cleanliness.

The shadow bugbear is with us a very real one, why it is not so with Dr. Jenkins I cannot understand, unless it is the perfection of joint is so good and that the polish of the inlay runs into and is in a measure continuous with the tooth.

